# Exploring the Multifaceted Potential of a Peptide Fraction Derived from *Saccharomyces cerevisiae* Metabolism: Antimicrobial, Antioxidant, Antidiabetic, and Anti-Inflammatory Properties

**DOI:** 10.3390/antibiotics12081332

**Published:** 2023-08-18

**Authors:** Patrícia Branco, Elisabete Muchagato Maurício, Ana Costa, Diogo Ventura, Catarina Roma-Rodrigues, Maria Paula Duarte, Alexandra R. Fernandes, Catarina Prista

**Affiliations:** 1 School of Engineering, Lusófona University, Campo Grande 376, 1749-024 Lisboa, Portugal; 2 Linking Landscape, Environment, Agriculture and Food (LEAF), Associated Laboratory TERRA, Instituto Superior de Agronomia, University of Lisbon, Tapada da Ajuda, 1349-017 Lisboa, Portugal; 3 Unit of Bioenergy and Biorefinary, Laboratório Nacional de Energia e Geologia (LNEG), Estrada do Paço do Lumiar, 22, 1649-038 Lisboa, Portugal; 4 CBIOS—Universidade Lusófona’s Research Center for Biosciences & Health Technologies, Campo Grande 376, 1749-024 Lisboa, Portugal; 5 Elisa Câmara, Lda, Dermocosmética, Centro Empresarial de Talaíde, n°7 e 8, 2785-723 São Domingos de Rana, Portugal; 6 UCIBIO—Applied Molecular Biosciences Unit, Department Ciências da Vida, NOVA School of Science and Technology, 2829-516 Caparica, Portugal; 7 i4HB, Associate Laboratory—Institute for Health and Bioeconomy, Faculdade de Ciências e Tecnologia, Universidade NOVA de Lisboa, 2829-516 Caparica, Portugal; 8 MEtRICs, Departamento de Química, NOVA School of Science and Technology|FCTNOVA, Campus de Caparica, Universidade Nova de Lisboa, 2829-516 Caparica, Portugal

**Keywords:** *Saccharomyces cerevisiae*, foodborne pathogens, antimicrobial peptides, bioactive metabolites, biopreservatives, antioxidant activity, antidiabetic activity, anti-inflammatory activity

## Abstract

The rising demand for minimally processed, natural, and healthier food products has led to the search for alternative and multifunctional bioactive food components. Therefore, the present study focuses on the functional proprieties of a peptide fraction derived from *Saccharomyces cerevisiae* metabolism. The antimicrobial activity of the peptide fraction is evaluated against various foodborne pathogens, including *Candida albicans*, *Candida krusei*, *Escherichia coli*, *Listeria monocytogenes*, and *Salmonella* sp. The peptide fraction antioxidant properties are assessed using FRAP and DPPH scavenging capacity assays. Furthermore, the peptide fraction’s cytotoxicity is evaluated in colorectal carcinoma and normal colon epithelial cells while its potential as an antidiabetic agent is investigated through α-amylase and α-glucosidase inhibitory assays. The results demonstrate that the 2–10 kDa peptide fraction exhibits antimicrobial effects against all tested microorganisms, except *C. krusei*. The minimal inhibitory concentration for *E. coli*, *L. monocytogenes*, and *Salmonella* sp. remains consistently low, at 0.25 mg/mL, while *C. albicans* requires a higher concentration of 1.0 mg/mL. Furthermore, the peptide fraction displays antioxidant activity, as evidenced by DPPH radical scavenging activity of 81.03%, and FRAP values of 1042.50 ± 32.5 µM TE/mL at 1.0 mg/mL. The peptide fraction exhibits no cytotoxicity in both tumor and non-tumoral human cells at a concentration up to 0.3 mg/mL. Moreover, the peptide fraction presents anti-inflammatory activity, significantly reducing the expression of the TNFα gene by more than 29.7% in non-stimulated colon cells and by 50% in lipopolysaccharide-stimulated colon cells. It also inhibits the activity of the carbohydrate digestive enzymes α-amylase (IC_50_ of 199.3 ± 0.9 µg/mL) and α-glucosidase (IC_20_ of 270.6 ± 6.0 µg/mL). Overall, the findings showed that the peptide fraction exhibits antibacterial, antioxidant, anti-inflammatory, and antidiabetic activity. This study represents a step forward in the evaluation of the functional biological properties of *S. cerevisiae* bioactive peptides.

## 1. Introduction

Various pathogenic microorganisms, including *E. coli*, *L. monocytogenes*, *Salmonella* spp., and *Candida* species, pose a risk of contaminating food products [1,2,3]. Foodborne diseases caused by microbial contamination are a significant global concern, leading to adverse health effects and substantial economic impacts such as increased public health expenses, food waste, and limitations on storage and transportation [4].

*E. coli*, commonly found in the gastrointestinal tract of humans and animals, is widely distributed in the environment. Consumption of contaminated food, such as meat, vegetables, and dairy products, is the primary mode of transmission for these bacteria [5]. While some strains of *E. coli* are harmless, other are pathogenic and can cause severe foodborne illnesses, including diarrhea, kidney failure, and even death [6].

*L. monocytogenes* is an opportunistic foodborne pathogen responsible for listeriosis, particularly affecting individuals with weakened immune systems [1]. It is commonly found in meat and dairy products, with cheese being an important source. *L. monocytogenes* can contaminate food at any stage of production, and it can survive and grow at refrigeration temperatures, posing a significant public health risk [7].

*Salmonella* sp. is typically detected in food products such as poultry, pork, milk, and eggs, but it can also originate from other sources, including dairy products, fruits, and vegetables [8,9]. Ingesting *Salmonella* can lead to a range of symptoms, including diarrhea, fever, and abdominal cramps. In severe cases, hospitalization and even death can occur, particularly among vulnerable populations such as young children and the elderly [10].

*C. albicans*, a yeast commonly present in the human body as a commensal microorganism, can become an opportunistic pathogen causing infections. While candidiasis is typically associated with mucocutaneous infections, recent studies have shown that *C. albicans* is able to survive and grow in several food products, including dairy, meat, and vegetables, posing risks to human health, and causing economic losses due to food spoilage [11,12,13].

*C. krusei*, known for its surface-growing film on foods, possesses characteristics that enable it to tolerate the conditions found in food products, resulting in spoilage. It is highly tolerant to low pH and high preservative concentrations, making acid-preserved food susceptible to spoilage, leading to excessive carbon dioxide production, bloating, and packaging ruptures [14,15]. Additionally, *C. krusei* has been reported as a cause of candidiasis, possibly due to its resistance to the antifungal agent fluconazole [16].

To mitigate the growth of these pathogenic microorganisms, prevent physical and chemical changes in food products, and extend their shelf life, the food industry commonly employs chemical preservatives. However, many of these preservatives, such as sulphur dioxide, sodium benzoate, benzoic acid, sodium sorbate, potassium sorbate, and sodium nitrite, pose risks to human health. Sulphur dioxide, for example, can cause headaches, palpitations, and allergies, while benzoates have been associated with allergies, asthma, and skin rashes. Sorbates and sorbic acid, though rarely, have been linked to urticaria and contact dermatitis [17,18,19].

Therefore, the increasing consumer interest in healthier foods, without chemical preservatives, is pressing the food industry towards less processed and more natural products, the so-called “clean label” products [20]. In response, the application of natural preservatives, such as bioactive metabolites produced by microorganisms, emerges as a compelling alternative strategy to develop healthier, “clean label”, food products while maintaining their safety and shelf life.

One alternative approach gaining traction involves the use of natural preservatives, such as bioactive metabolites produced by microorganisms. Numerous bioactive metabolites with antimicrobial activity, including antimicrobial peptides, bacteriocins, and mycocins, have been identified [21,22,23].

Certain strains of *S. cerevisiae* have been found to produce bioactive peptides, which exhibit antagonistic effects against some wine yeasts and lactic-acid bacteria [24,25,26,27]. In a previous work [25], a 2–10 kDa peptide fraction obtained from *S. cerevisiae* strain CCMI 885 supernatant was fractionated by gel-filtration chromatography and four peaks were obtained and collected for antimicrobial assays. Peak II, with an apparent MW of 8 kDa, exhibited antagonistic effects against some wine yeasts and lactic acid bacteria and was found to contain bioactive peptides derived from the glyceraldehyde 3-phosphate dehydrogenase enzyme [25]. Afterward, Branco et al. (2017) [26] screened eight *S. cerevisiae* strains, including the one used in this study (ISA 1028), for their production of peptides. The results of 2–10 kDa fractions gel-filtration chromatography, obtained from each strain supernatant, showed that all *S. cerevisiae* strains presented a similar chromatographic profile, with four peaks ranging from approximately 2 to 10 kDa [26]. A correlation between the peak II area and the antagonistic effect of each *S. cerevisiae* strain against one wine yeast was detected, revealing that *S. cerevisiae* CCMI 885 and *S. cerevisiae* ISA 1028 were the strains with the higher antagonistic effect against *Hanseniaspora guilliermondii*. Similarly, Al-Sahlany et al. (2020) [28] fractioned, by gel-filtration chromatography, a 2–10 kDa peptide fraction obtained from *S. cerevisiae* strain ATCC 36858 supernatant and found a peptide with a molecular weight of 9.77 kDa that inhibits the growth of *E. coli* and *Staphylococcus aureus.*

Bioactive peptides have a wide range of functional properties, including antimicrobial, anticancer, antihypertensive, antidiabetics, antioxidative, and anti-inflammatory activity [29]. For instance, Mudgil et al. (2018) [30] discovered that peptides derived from camel milk protein hydrolysates demonstrated antidiabetic and anti-obesity properties. Likewise, a peptide derived from mushroom *Ganoderma lucidum* exhibited antioxidant activity and exerted antimicrobial effects against *E. coli* and *Salmonella typhi* [31,32,33].

Antioxidants are particularly important in preventing the oxidation of compounds and are found abundantly in natural foods such as fruits and vegetables; they include polyphenols, such as flavonoids [34,35,36,37], and bioactive peptides [38]. In addition, they have been found in yeast peptide fractions and yeast extracts [39,40]. The consumption of antioxidant-rich foods has been associated with numerous health benefits [41]. Antioxidants play a vital role in safeguarding cells against oxidative stress caused by free radicals, which have been linked to various chronic diseases such as cancer, heart disease, and Alzheimer’s disease [41,42,43].

Taking into account the previous findings on the 2–10 kDa fraction derived from *S. cerevisiae* strains metabolism and all the possible functional properties previously detected on bioactive peptides, the aim of this study is to assess the functional properties of the 2–10 kDa peptide fraction derived from *S. cerevisiae* strain (ISA 1028) metabolism, namely its (i) antimicrobial activity against foodborne pathogens, (ii) potential as natural antioxidants, (iii) anti-inflammatory activity on human colon epithelial cells, and (iv) antidiabetic activity.

## 2. Results

### 2.1. Antimicrobial Activity of the 2–10 kDa Fraction against FOODBORNE Pathogens

#### 2.1.1. Minimal Inhibitory Concentration (MIC)

The antimicrobial activity of the peptide fraction (2–10 kDa) derived from the fermentation supernatant of *S. cerevisiae* strain ISA 1028 was evaluated by determining the MIC against three bacterial strains (*E. coli* ATCC 25922, *L. monocytogenes* ISA 4008, and *Salmonella* sp. ISA 4348) and two yeast strains *(C. albicans* ISA 2289 and *C. krusei* ISA 2290).

The analysis of the peptide fraction by High-Performance Liquid Chromatography (HPLC) revealed the presence of ethanol (8% (*v*/*v*)). Hence, the maximum concentration of ethanol in the MIC assay is 4% (*v*/*v*), since the 2–10 kDa fraction is mixed with the growth medium in a proportion of 1:1 and serial dilutions of them were made in the microplate according to the assay methodology.

The results confirmed that all tested microorganisms exhibited normal growth in the absence of the peptide fraction and in the presence of ethanol (negative control). In addition, the microorganisms under trial were also tested against suitable antibiotics (positive controls) to ensure the proper sensitivity of each strain.

The MIC of the 2–10 kDa fraction against the three bacterial strains was 0.25 mg/mL, indicating that the antimicrobial peptide effectively inhibited the growth of these bacteria at a relatively low concentration (Table 1).

On the other hand, the MIC of the 2–10 kDa fraction against *C. albicans* was 1.0 mg/mL, indicating that a higher concentration is necessary to inhibit the growth of this pathogenic yeast when compared to the tested bacteria. (Table 1). However, the MIC of the 2–10 kDa fraction against *C. krusei* was higher than 1.0 mg/mL, indicating that this particular pathogenic yeast resisted the 2–10 kDa fraction at the tested concentrations (Table 1). Considering that several authors classify the antimicrobial activity of natural substances as strong inhibitors (MIC up to 0.5 mg/mL), moderate inhibitors (MIC between 0.6 and 1.5 mg/mL), and weak inhibitors (MIC above 1.6 mg/mL) [44,45,46], it is possible to confirm that the peptide fraction under study presents a strong inhibitory activity against bacteria and moderate against the *C. albicans*.

#### 2.1.2. Fungicidal and Bactericidal Activity

To assess the bactericidal and fungicidal activity of the 2–10 kDa fraction against the microorganism showing higher sensitivity to these peptides, i.e., *C. albicans*, *E. coli*, *L. monocytogenes* and *Salmonella* sp., we studied the viability of the microorganisms by counting colony-forming units per milliliter (CFU/mL). Samples from the negative control and test assays at the MIC value (0.25 mg/mL for bacteria and 1.0 mg/mL for *C. albicans*) and at concentrations higher than the MIC were taken after 24 h and colony-forming units were counted.

**Figure 1:**
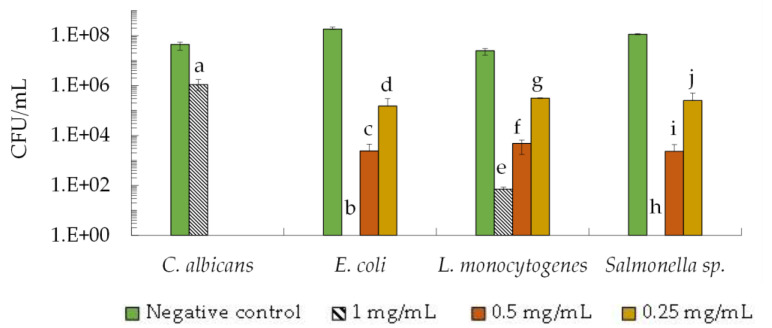
Viability (CFU/mL), of *C. albicans* ISA 2289, E. coli ATCC 25922, *L. monocytogenes* ISA 4008 and *Salmonella* sp. ISA in the presence of fraction 2–10 kDa at a final concentration of 1.0, 0.5 and 0.25 mg/mL and in the absence of fraction 2–10 kDa (Negative control). Data represented correspond to means ± SD (error bars) of three independent biological assays. Different letters (a–j) represent *p*-value < 0.05 relative to respective control.

The results demonstrated that in the absence of the 2–10 kDa fraction (negative control), all microorganisms reached a viability of 10^7^ CFU/mL to 10^8^ CFU/mL within 24 h (Figure 1). When the 2–10 kDa fraction was present at the MIC value (0.25 mg/mL), the viability of bacteria decreased by 2 to 3 orders of magnitude when compared to the negative control without the peptide fraction. Specifically, the CFU/mL values were 1.5 × 10^5^ CFU/mL (*E. coli*), 2.5 × 10^5^ CFU/mL (*Salmonella* sp.), and 3.2 × 10^5^ CFU/mL (*L. monocytogenes*) (Figure 1).

As expected, the peptide fraction exhibited increased antimicrobial activity at a higher concentration of 0.5 mg/mL, resulting in a decrease of 4 to 5 orders of magnitude in CFU/mL when compared to the control-assay values. The CFU/mL values at this concentration were 2.9 × 10^3^ CFU/mL (*E. coli*), 2.3 × 10^3^ CFU/mL (*Salmonella* sp.) and 4.7 × 10^3^ CFU/mL (*L. monocytogenes*) (Figure 1). Interestingly, at the highest concentration tested (1.0 mg/mL), no growth was observed for *E. coli* and *Salmonella* sp., while *L. monocytogenes* showed a partial inhibition with a viability of 70 CFU/mL (Figure 1). Once again, *C. albicans* demonstrated greater resistance to the peptide fraction compared to bacteria, as a viability of 1.0 × 10^6^ CFU/mL was detected when cells were exposed to 1.0 mg/mL of the 2–10 kDa fraction (Figure 1).

### 2.2. Antioxidant Activity of 2–10 kDa Fraction

**Figure 2:**
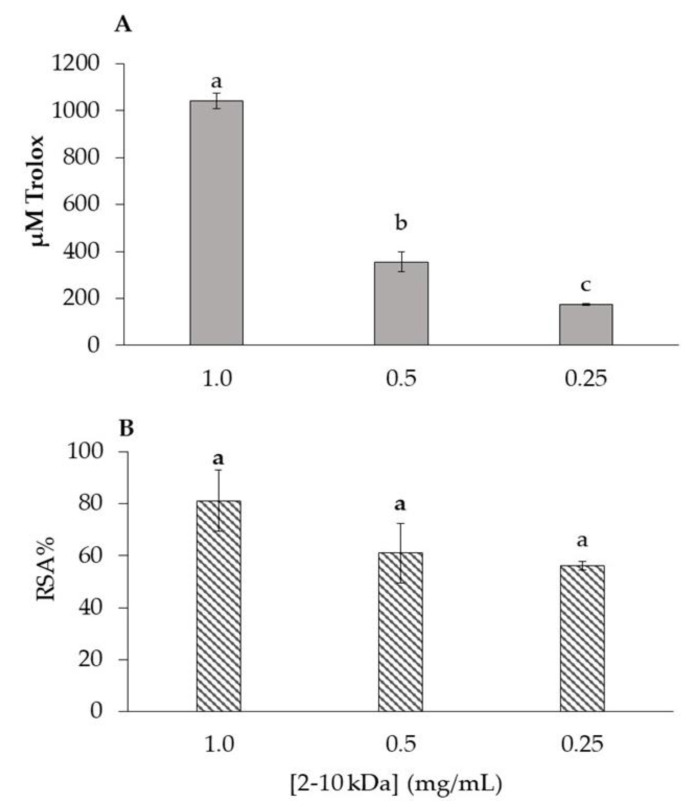
Values obtained for FRAP expressed in µM Trolox (**A**) and for DPPH expressed in RSA% (**B**), for the 2–10 kDa fraction at 1.0, 0.5 and 0.25 mg/mL. Different letters (a–c) represent significant differences (*p* < 0.05).

The antioxidant capacity of the 2–10 kDa peptide fraction at 1.0, 0.5, and 0.25 mg/mL was determined by two methods, one scavenging based assay (DPPH radical scavenging activity (RSA%)) and one measuring reducing power (Ferric Reducing Antioxidant Power (FRAP)). Trolox was used as the standard for the DPPH and FRAP assays (Figure S1 depicts the calibration curve for each assay), and the results are represented in Figure 2. In the case of FRAP assay (Figure 2A), the fraction at 1.0 mg/mL showed the highest antioxidant capacity (1042.50 ± 32.5 µM TE/mL). With the fraction concentration reduction to 0.5 mg/mL, a reduction to 355.00 ± 42.5 µM TE/mL is also observed (Figure 2A). The same phenomenon occurs with the 2–10 kDa fraction at 0.25 mg/mL, in which the presented value also decreases around 50% (173.75 ± 3.75 µM TE/mL) (Figure 2A). Among the concentrations tested, the 2–10 kDa fraction at 1.0 mg/mL concentration had the highest capacity to inhibit DPPH radical with a %RSA of 81.03%, while at 0.5 mg/mL it was 60.99% and at 0.25 mg/mL was 56.22% (Figure 2B).

### 2.3. Cytotoxicity of 2–10 kDa Fraction

**Figure 3:**
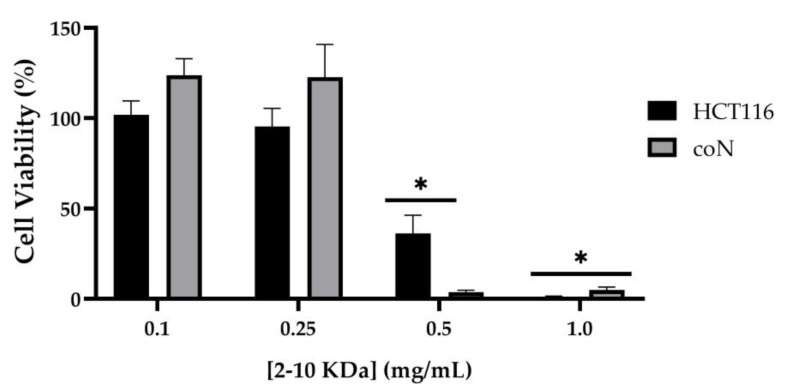
Cytotoxicity of peptide fraction 2–10 kDa in colorectal carcinoma cells (HCT116) and normal colon cells (coN). Cells were incubated for 48 h with crescent concentrations of the peptide fraction 2–10 kDa and cell viability was measured through the MTS assay. Bars represent the average ± SEM of four experiments. * *p*-value < 0.05 relative to respective control.

To assess if the 2–10 kDa fraction presents cytotoxicity in human cells, the viability of normal colon epithelial cells (coN) and colorectal carcinoma cells (HCT116) was evaluated after 48 h incubation with increasing concentrations of the peptide fraction. Results showed that the fraction presented no cytotoxicity for concentrations up to 0.25–0.30 mg/mL. Moreover, independently of the tumorigenic characteristic of the cells, an IC_50_ of 0.4 mg/mL was observed in colon epithelial cells (Table 2, Figure 3). Importantly, at the MIC concentration for antibacterial activity (0.25 mg/mL, Table 1), there is no significant impact on the viability of the human cells (100% activity).

### 2.4. Analysis of Anti-Inflammatory Effect of Peptide Fraction 2–10 kDa

To understand if the peptide fraction has some effect on the inflammatory process, coN cells were exposed to an inflammatory stimulus (lipopolysaccharides (LPS)) and then incubated for 3 h with 0.25 mg/mL of the peptide fraction 2–10 kDa or 1% (*v*/*v*) ethanol (as the vector control). Cells that were not exposed to the inflammatory stimulus were used for comparison.

**Figure 4:**
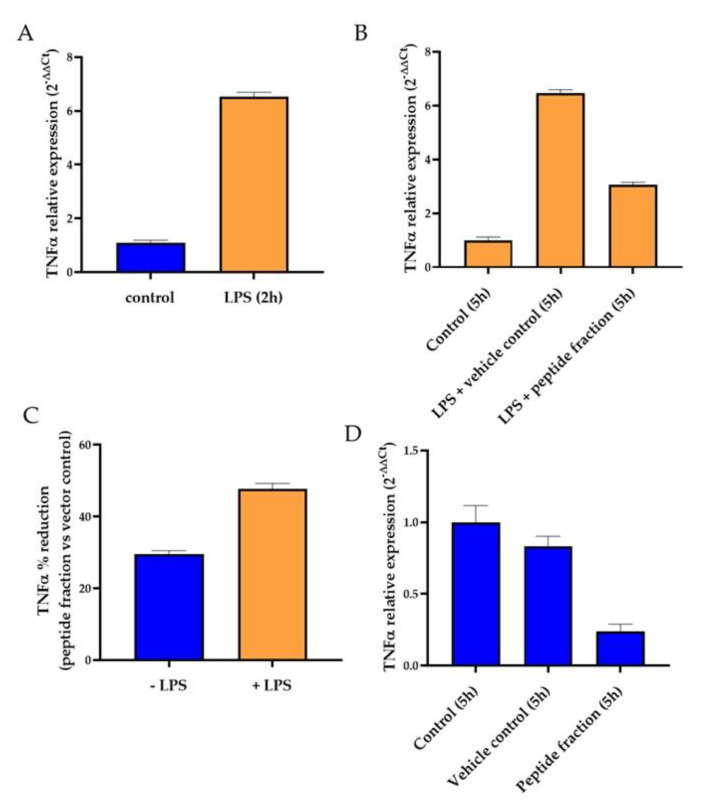
Effect of peptide fraction 2–10 kDa in TNF-α expression in colon epithelial cells (coN). Cells were incubated for 2 h with 7 µg/mL lipopolysaccharide (+LPS) and then for 3 h with ethanol 1% (*v*/*v*) or 0.25 mg/mL peptide fraction 2–10 kDa. In parallel, cells were submitted to the same treatment but without LPS (-LPS, (**A**)) Gene expression after 2 h in +LPS samples (orange bars), calculated by 2^−∆∆Ct^, using as reference RNA 18S gene and—LPS samples (blue bars, (**B**)). Gene expression in LPS treated samples and exposed to vehicle control and peptide fraction calculated by 2^−∆∆Ct^, using as reference RNA 18S gene and samples only treated with LPS for 5 h, (**C**). Percentage of TNF-α reduction, after incubation with LPS (orange bars) or without LPS exposure (blue bars, (**D**)). Gene expression in LPS untreated samples (-LPS) and exposed to vehicle control and peptide fraction calculated by 2^−∆∆Ct^, using as reference RNA 18S gene and untreated samples collected after 5 h. Error bars represent SEM of at least three independent experiments. *p*-value  <  0.05 relative to respective control sample (treated with ethanol).

The inflammatory potential was evaluated by RT-qPCR, measuring the expression levels of the gene encoding the pro-inflammatory cytokine tumor necrosis factor alpha (TNF-α) (Figure 4). The analysis of TNF-α expression on coN cells after 2 h incubation in the presence of LPS confirmed the induction of the cytokine expression when cells are exposed to an inflammatory stimulus (Figure 4A). After additional 3 h of incubation in the presence of LPS, TNF-α expression remained high (Figure 4B). However, a 52% reduction of TNF-α expression was observed when LPS-treated colon cells were incubated in the presence of the peptide fraction 2–10 kDa when compared to the cells only treated with LPS (Figure 4B,C (+LPS, orange bars)), demonstrating the anti-inflammatory capability of the peptide fraction. Moreover, this anti-inflammatory potential is also observed even in the absence of the LPS stimulus (−LPS) but to a lower extent (only a 29% reduction of TNF-α expression was observed) (Figure 4C,D, blue bars).

### 2.5. Antidiabetic Effect of Peptide Fraction 2–10 kDa

The antidiabetic potential of the peptide fraction was evaluated by its in vitro α-amylase and α-glucosidase inhibitory activities through colorimetric methods. Acarbose, an antidiabetic drug that acts by inhibiting α-amylase and α-glucosidase activities, was used as a positive control (Table 3).

The peptide fraction inhibited both enzymes in a dose-dependent way. However, an inhibitory activity lower than the positive control (acarbose) was observed. The inhibitory activity of the peptide fraction was more pronounced against α-amylase. In fact, under the experimental conditions used, the peptide fraction totally inhibited the α-amylase activity, making it possible to determine the IC_50_ value i.e., the concentration that inhibits 50% of the enzyme activity under the specific set of assay conditions. In what concerns α-glucosidase, under the experimental conditions used, only a maximum inhibition of about 20% was verified, whereby the IC_20_ value was determined.

## 3. Discussion

Microbiological contamination in food products is a global concern due to the increasing incidence of foodborne diseases and their significant economic impact, including limitations on storage and transport time, increased public health expenses, and food waste. Therefore, it is crucial to prevent and control the growth of spoilage microorganisms. Foodborne pathogens such as *L. monocytogenes*, *E. coli*, *Salmonella* sp., and *Candida* sp. pose a significant food safety risk and can cause illnesses [3,4]. The use of chemical preservatives to control the growth of these microorganisms is a common practice. However, these preservatives can adversely affect human health [47]. Hence, there is a need to explore alternative preservatives that are both less toxic and can effectively eliminate and prevent the proliferation of foodborne pathogens in food products. In fact, some antimicrobial peptides (AMPs) produced by bacteria that are classified as Generally Recognized As Safe (GRAS) [48] were applied as food preservatives, such is the case of nisin [49,50,51].

Nisin, a bacteriocin derived from the bacterium *Lactococcus lactis*, has emerged as a promising natural preservative in the food industry. Its antimicrobial activity has been extensively studied, demonstrating efficacy against a wide range of spoilage and pathogenic microorganisms, including Gram-positive bacteria and some Gram-negative bacteria [52,53]. Nisin has been successfully utilized in various food products, including dairy, meat, poultry, bakery, and beverages, to inhibit the growth of spoilage and pathogenic microorganisms, thus extending the shelf life and enhancing food safety [54].

In addition to bacteriocins, yeasts produce extracellular proteins known as mycocins, which exhibit antimicrobial activity [55]. Research conducted by Izgü et al. (2007) [56] demonstrated that *Wickerhamomyces anomalus* NCYC 434 secretes a mycocin that effectively inhibits *C. krusei*. Similarly, Al-Qaysi et al. (2017) [57] found that *Debaromyces hansenii* DSMZ 70238 secretes a mycocin with antagonistic effects against *Candida* spp., *E. coli*, and *S. aureus*. Furthermore, mycocins secreted by *Tetrapisispora phaffii* DBVPG 6706 and *W. anomalus* DBVPG 3003 have demonstrated activity against various wine yeasts, including strains of *Brettanomyces bruxellensis*, which is a major cause of wine spoilage worldwide [58,59,60].

Another yeast well known for its ability to produce several bioactive compounds and metabolites, including peptides, carbohydrates such β-glucans and mannans, vitamins and antioxidant enzymes, is *S. cerevisiae* [61,62]. Previous studies [24,25,26,27,28] have identified the presence of antimicrobial activity within a peptide fraction ranging from 2–10 kDa derived from *S. cerevisiae* metabolism. This fraction contains antimicrobial peptides that play a crucial role in inhibiting the growth of some yeast, i.e., *Hanseniaspora guilliermondii*, *Lachancea thermotolerans*, *Torulaspora delbrueckii*, *Kluyveromyces marxianus, B. bruxellensis*, and bacteria such as lactic acid bacteria [24,25,26,27], *E. coli*, and *S. aureus* [28].

In this study, for the first time, the antimicrobial activity of the 2–10 kDa fraction derived from the metabolic processes of *S. cerevisiae* was tested against *L. monocytogenes*, *Salmonella* sp., and *Candida* species as well as its functional proprieties, i.e., antioxidant, anti-inflammatory, and antidiabetic effects.

The antimicrobial assays demonstrated that the 2–10 kDa fraction had an inhibitory effect on all the tested microorganisms, except for the yeast *C. krusei* (Table 1, Figure 1), suggesting an inherent resistance of this pathogenic yeast to the peptide fraction.

It can be observed that the fraction exhibits a significant antibacterial activity against the Gram-positive (*L. monocytogenes*) and Gram-negative (*Salmonella* sp. and *E. coli*) bacteria used in this study (Table 1, Figure 1). These findings show that the fraction under study has a broad spectrum of action and thus demonstrates potential to control foodborne pathogens and food spoilage. Moreover, the results highlight the advantage of these peptides over other natural antimicrobials compounds, namely some natural extracts rich in polyphenols that are typically more specific towards Gram-positive bacteria [37,63,64].

Several factors influence the antioxidant capacity of peptides, including their amino acid composition, sequence, and hydrophobic amino acid content [65,66,67]. As can be seen from Figure 2, the 2–10 kDa fraction at the different concentrations hold antioxidant capacity. The differences between DPPH and FRAP results may result from to their underlying principles. The DPPH assay is based on the measurement of the scavenging capacity of antioxidants towards it [68] and the FRAP assay is based on the compounds capability to reduce Fe^3+^ to Fe^2+^ [69], which may justify the lowest results in the case of FRAP. However, while for the DPPH assay there were no significant differences between the concentrations, in the case of the FRAP assay, a significant correlation with the peptide concentration was observed, which may indicate that, for DPPH, other factors besides peptides can be disturbing the results. Particularly, the concentration of 1.0 mg/mL stood out, while the concentrations of 0.5 and 0.25 mg/mL showed very similar values.

Mirzaei et al. (2015) [61] conducted a study to assess the antioxidant activity of different peptide fractions derived from *S. cerevisiae* using the DPPH inhibition assay. The study examined peptide fractions with varying molecular weights (>3 kDa, 3–5 kDa, 5–10 kDa, and <10 kDa). The results showed that the <3 kDa fraction obtained from trypsin isolate exhibited the highest antioxidant capacity, measuring 489.12  ±  0.001 µM TE/mg protein. In a more recent study by Mirzaei et al. (2021) [67], the researchers performed the FRAP assay in order to evaluate the antioxidant potential of the peptide fractions. Interestingly, the <3 kDa fraction showed a lower result than the 3–5 kDa fraction. It is worth mentioning that the antioxidant activity observed in this current study, using both the DPPH and FRAP assays, was higher than the values reported by the author in their previous work.

While the use of bioactive metabolites as food preservatives shows to be great promise, it is crucial to ensure that these metabolites are safe for consumption and do not have any adverse effects on human health. Studies investigating the cytotoxicity of bioactive metabolites such as antimicrobial peptides on gastrointestinal tract cell lines have shown minimal or no cytotoxic effects at concentrations effective for controlling microbial growth [70]. Therefore, and for the first time, the biological effect of the 2–10 kDa fraction derived from *S. cerevisiae* metabolism was evaluated in human cell cultures, namely in colorectal carcinoma cells (HCT116) and normal colon cells (coN). Regarding the biological effect of the 2–10 kDa fraction in human cells, it was observed that the two tested cell cultures presented an IC_50_ higher than 0.4 mg/mL (Table 2), with 100% viability at 0.3 mg/mL. This result suggests the 2–10 kDa fraction possesses antibacterial activity (MIC 0.25 mg/mL) in concentrations lower than the IC_50_ obtained in this study. These findings emphasize the potential application of the peptide fraction as an antibacterial or as co-adjuvant preservative in food or in health care. Moreover, the peptide fraction showed an anti-inflammatory capability as evidenced by its impact on the expression of TNF-α, regardless of the presence or absence of the inflammatory stimulus, LPS. However, this anti-inflammatory potential was higher when coN cells were previously stimulated with LPS (Figure 4). Overall, these results suggest that the 2–10 kDa fraction possesses an anti-inflammatory potential in colon cells, independent of their previous stimulation with an inflammatory agent, although this response is more pronounced when cells were previously exposed to the inflammatory stimulus.

Type 2 diabetes is a major public health concern, characterized by persistent hyperglycemia coupled with several metabolic dysfunctions. Inhibiting the activity of the carbohydrate digestive enzymes to avoid the degradation of polysaccharides into glucose is one approach to prevent postprandial hyperglycemia. Therefore, α-amylase and α-glucosidase inhibitors have been considered first-line drugs to control blood sugar levels, thus helping to prevent and control type 2 diabetes [71]. Some currently available antidiabetic drugs, such as acarbose, act by inhibiting the activity of these two enzymes. However, these drugs are associated with gastrointestinal disturbance, abdominal pain, and flatulence [72]. Thus, many studies have focused on identifying alternative α-amylase and α-glucosidase inhibitors, such as plant extracts [73] or food derived bioactive peptides [30,71,72,74,75,76], that may simultaneously have inhibitory activity and a low incidence of undesirable side effects.

The peptide fraction was able to inhibit the activity of α-amylase and α-glucosidase, the effect being more pronounced towards α-amylase. Other peptides have been shown to inhibit α-amylase and α-glucosidase, with IC_50_ values within the range of 0.027–23.30 mg/mL and 1.45–10 mg/mL, respectively [30,71,74,75,76], with these inhibitory activities related to their amino acid composition. Some authors suggested that branched chain, such as isoleucine, aromatic, such as tyrosine and tryptophan, and positively charged, such as arginine, residues are preferably bound to α-amylase and could be key amino acids associated with α-amylase inhibitory activity [72,74]. On the other hand, the presence of basic amino acids at the N-terminal and of proline within the chain and alanine or methionine at the C-terminal seems to be important for the α-glucosidase inhibitory activity [72].

## 4. Materials and Methods

### 4.1. Production of the 2–10 kDa Fraction

The peptide fraction was obtained as described in [25]. Briefly, *S. cerevisiae* ISA 1028 cells were grown at 25 °C, without agitation in Synthetic Grape Juice at pH 4.5 (glucose 110 g/L, fructose 110 g/L; acids solution: tartaric acid 6 g/L, malic acid 3 g/L, citric acid 0.5 g/L; amino acids solution: yeast nitrogen base without aa 1.7 g/L, casamino acids 2 g/L, calcium chloride 0.2 g/L, arginine 0.8 g/L, proline 1 g/L, tryptophan 0.1 g/L and 2.5 g/L yeast extract). All solutions were autoclaved, except the amino acid’s solution, which was sterilized by filtration (0.22 µm). After seven days, cells were removed by centrifugation and the supernatant was first filtered through a 0.45 µm membrane and then through a 0.22 µm membrane (Merck Millipore, EUA, Burlington, MA, USA). Then, the obtained cell-free supernatant was first ultrafiltrate through centrifugal filter units (Vivaspin 15R, Sartorius, Göttingen, Germany) equipped with 10 kDa membranes and then concentrated (40-fold) with 2 kDa membranes, obtaining a 2–10 kDa fraction. Ethanol and organic acids present in the 2–10 kDa fraction were analysed in duplicate using a High-Performance Liquid Chromatography (HPLC) VWR Hitachi Chromaster system (VWR, Radnor, PA, USA) equipped with a refractive index detector (Chromaster HPLC 5310) and UV-Vis detector (Chromaster HPLC 5420) (Waters, Dublin, Ireland) equipped with a refractive index detector (2414Waters). The 2–10 kDa fraction was first filtrated by 0.22 μm Millipore membranes (Merck Millipore, EUA, Burlington, MA, USA) and then injected in a Rezex™ ROA Organic Acid H+ (8%) column (300 mm 7.8 mm, Phenomenex, Torrance, CA, USA) and eluted with sulfuric acid (5 mmol/L) at 65 °C with a flow rate of 0.5 mL/min.

### 4.2. Antimicrobial-Assays

The antimicrobial activity was determined using the broth microdilution method, according Clinical and Laboratory Standards Institute guidelines [75]. All assays were performed in triplicate, and positive (chloramphenicol for bacteria or ketoconazole for yeasts) and negative (ethanol) controls were included in all assays.

#### 4.2.1. Microorganisms and Media

In this work, the following microorganisms from Culture collection of Instituto Superior de Agronomia, Portugal, were used: *C. albicans* ISA 2289; *C. krusei* ISA 2290; *L. monocytogenes* ISA 4008; *S. cerevisiae* ISA 1028, *Salmonella* sp. ISA 4348, and *E. coli* ATCC 25922 from the American Type Culture Collection. The antimicrobial activity was assessed against the microorganisms described above. In order to prepare the working culture of the microorganisms, a subculture was prepared from the stock culture in plates with recommended media and incubation conditions for each microorganism, Luria-Bertani (LB) agar plates (10 g/L tryptone, 10 g/L NaCl, 5 g/L yeast extract, 20 g/L agar, pH was adjusted to 7.0 with 5 N NaOH) for *E. coli* ATCC 25922 and *Salmonella* sp. ISA 4348 and Brain Heart Infusion (BHI) (Biokar Diagnostics, Allonne, France) for *L. monocytogenes* ISA 4008 at 35 ± 2 °C, or Sabouraud Dextrose Agar (SDA) (Biokar Diagnostics, Allonne, France) for yeasts at 30 ± 2 °C [37].

#### 4.2.2. Determination of Minimum Inhibitory Concentrations

The conventional minimal inhibitory concentration (MIC) was determined by the broth microdilution method in 96-well microtiter plates, according to Pereira et al. (2022) [46]. The peptide fraction was prepared at 2 mg/mL and a dilution of 1:2 was introduced in the first line, followed by a series of 2-fold dilutions in Sabouraud Dextrose broth (Biokar Diagnostics, Allonne, France) for yeasts (*C. albicans* and *C. krusei*) and in Mueller-Hinton broth (Biokar Diagnostics, Allonne, France) for the remaining bacteria. Ethanol at 8% (*v*/*v*) was used as negative control and positive controls, 100 mg/L of chloramphenicol (Merck KGaA, Darmstadt, Germany) for bacteria or 0.1 mg/mL of ketoconazole (Merck KGaA, Darmstadt, Germany) for yeasts, were used in the same conditions and dilutions.

To inoculate the 96-well microtiter plates, a standardized saline suspension (NaCl, 0.85% *w*/*v*) was prepared, and turbidity adjusted to 0.5 McFarland scale (DEN-1, McFarland Densitometer, Biosan) corresponding to 1–2 × 10^8^ CFU/mL for bacteria and 1–5 × 10^6^ CFU/mL for yeasts. The inoculation was performed according to the CLSI document M07-A9, 2012 [77]. The microplates were then incubated at 35 °C ± 2 for all microorganisms, for 24 h, except for yeasts, which were incubated for 24 h at 30 °C ± 2. After the incubation period, microplates were visually observed to determine the MIC (defined as the lowest peptide fraction concentration at which no visible growth could be detected) and samples were taken to evaluate the fungicidal and bactericidal activity.

#### 4.2.3. Evaluation of Fungicidal and Bactericidal Activity

After the incubation period (24 h) the target microorganisms’ culturability (CFU/mL) was determined by the classical plating method. Briefly, 100 μL of cells were plated onto LB agar plates (*E. coli* and *Salmonella* sp.), onto BHI agar (*L. monocytogenes*) or SDA plates (*C. albicans*) after appropriate dilution (decimal serial dilution method). Plates were incubated at 35 ± 2 °C (bacteria) and 30 ± 2 °C (yeasts) and the number of CFU enumerated after 1–2 days.

### 4.3. Antioxidant Activity

#### 4.3.1. Ferric Reducing Antioxidant Power (FRAP)

The FRAP assay is based on the compound’s capability to reduce Fe^3+^ to Fe^2+^, which will translate into antioxidant activity. A version of the Benzie and Strain (1996) [69] assay was used with modifications. The FRAP reagent utilized was composed of 25 mL of 0.3 M of acetate buffer (C_2_H_3_NaO_2_·3H_2_O, Merck KGaA, Darmstadt, Germany) pH 3.6, 2.5 mL of TPTZ 10 mM (2,4,6-tripyridyl-S-triazine, Alfa Aesar, MA, EUA) in 40 mM HCl and 2.5 mL of 20 mM ferric (III) chloride hexahydrate (FeCl_3_·6H_2_O, Sigma, St. Louis, MO, USA) at room temperature. In a 96 well microplate, 10.3 µL of sample diluted 1:10, 30.9 µL of ultrapure water (Merck KGaA, Darmstadt, Germany) and 309 µL of the FRAP reagent were added. It was then incubated in a MOBI microplate reader (µ2 MicroDigital Co., Ltd., Seoul, Republic of Korea) at 37 °C for 45 min. The absorbance was read at 595 nm before and after incubation. The blank was made with ultrapure water (Merck kGaA, Darmstadt, Germany) to substitute the sample. A calibration curve was made with TROLOX 2000 µM in concentrations of 0, 100, 200, 400, 500, 600, 750, 100, 1500, and 2000 µM. The results were expressed in µM.

#### 4.3.2. DPPH Radical Scavenging Assay (2,2-Diphenyl-1-picrylhydrazyl)

DPPH (2,2-Diphenyl-1-picrylhydrazyl, TCI, Tokyo, Japan) is a free radical that absorbs at 515 nm in its radical form but not in its reduced form. The protocol used was a modified version of Brand–Williams (1995) [68]. The DPPH 0.24 mg/mL solution was made with 0.6 mg of DPPH diluted in ethanol 100% (Aga, Lisbon, Portugal) at room temperature. In a 96 well microplate, 8.75 µL of sample and 341.25 µL of DPPH solution were added and incubated in a MOBI microplate reader (µ2 MicroDigital Co., Ltd., Republic of Korea) at 30 °C for 45 min. The absorbance was read at 515 nm before and after incubation, and the blank was made with ethanol 100% (Aga, Lisbon, Portugal) to replace the sample. The absorbance of the resulting solution was measured at 515 nm and radical-scavenging activity (%) was calculated as follows: Radical-scavenging activity (%) = (Absorbance_control_ − Absorbance_sample_ × 100)/Absorbance_control_). A calibration curve was made with TROLOX 2000 µM in concentrations of 0, 100, 200, 400, 500, 600, 750, 100, 1500 and 2000 µM. The results were expressed in µM.

### 4.4. Cytotoxicity Assay

The cytotoxic effect of peptide fraction 2–10 kDa was determined in colorectal carcinoma cells (HCT116, CCL-247 ATCC, Manassas, VA, USA) and colon epithelial cells (coN, CRL-1790, ATCC) using Dulbecco’s modified Eagle medium (DMEM, ThermoFisher Scientific, Waltham, MA, USA) supplemented with 10% (*v*/*v*) fetal bovine serum (FBS, ThermoFisher Scientific) and a mixture of 100 U/mL Penicilin and 100 µg/mL Streptomycin (ThermoFisher Scientific). Cells were seeded in a density of 7500 cells/well in a 96-well plate and after 24 h, submitted to crescent concentrations of peptide fraction 2–10 kDa or crescent concentrations of ethanol (vector control). After 48 h incubation at 37 °C, 5% (*v*/*v*) CO_2_ and saturated humidity, the medium was removed and replaced by a mixture of medium with Cell Titer 96^®^ Aqueous One solution cell proliferation assay (Promega, Madison, WI, USA) and protocol followed the manufacturer’s instructions. The viability percentage of cells exposed to each concentration of peptide fraction was normalized to the viability percentage of cells exposed to respective to the concentration of ethanol (vector control).

### 4.5. Inflammatory Effect

The anti-inflammatory potential of the peptide fraction 2–10 kDa was examined in coN cells with few alterations from what was previously published [78]. Fibroblasts and coN cells were seeded in 25 cm^2^ T-flasks and then incubated for 24 h at 37 °C, 5% (*v*/*v*) CO_2_ and 99% (*v*/*v*) relative humidity. Cells were incubated for 2 h with 7 µg/mL lipopolysaccharide (LPS, Sigma Aldrich, St. Louis, MO, USA), then 0.25 mg/mL peptide fraction 2–10 kDa or 1% (*v*/*v*) ethanol were added, and cells incubated for further 3 h. Similar samples without LPS were also prepared in parallel. Samples treated and untreated with LPS (+LPS and −LPS, respectively) were collected after 2 h incubation with LPS, and +LPS and −LPS samples incubated with peptide fraction or respective negative control were collected after 3 h (5 h since the beginning of the experiment) by cell detachment with Tryple Express (ThermoFisher Scientific), centrifugation at 500× *g* and pellet resuspension in NZYol (NZYtech, Lisbon, Portugal). RNA was extracted according to manufacturer’s instructions, cDNA synthesized using the NZY M-MULV First strand cDNA synthesis kit (NZYtech), and TNF-α and RNA 18S genes were amplified using the NZY Supreme qPCR Green Master Mix (NZYtech) in a Corbett-Rotor Gene thermal cycler (Qiagen, Hilden, Germany). The expression of TNF-α in samples was determined with the 2^−∆∆Ct^ method [79].

### 4.6. α-Amylase Inhibitory Assay

The inhibition of the α-amylase was determined according to the procedure described by Romeiras et al. (2023) [78]. Reaction mixtures with 100 μL of Type VI-B porcine pancreatic α-amylase (0.5 mg/mL in 100 mM sodium phosphate buffer, containing 6.7 mM sodium chloride, pH 6.7) and 100 μL of the peptide fraction 2–10 kDa (concentrations up to 1.53 mg/mL) were incubated at 37 °C for 10 min. Then, 100 μL of 1% starch (*w*/*v*), in 100 mM sodium phosphate buffer, containing 6.7 mM sodium chloride, pH 6.7) was added. After 10 min at 37 °C, 200 μL of DNS reagent (20 mL of 96 mM DNS, 8 mL of 5.315 M sodium potassium tartrate tetrahydrate in 2 M NaOH and water up to 40 mL) was added. The reaction mixtures were boiled at 100 °C for 15 min, cooled until room temperature, diluted with 2 mL of water and the absorbance (Abs) was measured at 520 nm (SPEKOL 1500, Analytik, Jena, Germany). The reaction mixture without peptide fraction was used as negative control (NC) and reaction mixtures prepared with inactive α-amylase were used as samples’ blanks (SB). Acarbose was used as positive control. The enzyme inhibitory rate was calculated as follows:

Inhibition (%) = [(Abs_NC_ − (Abs_sample_ − Abs_SB_) × 100/(Abs_NC_)]

Values were assessed in triplicate and the results were expressed as the final concentration (μg/mL), in the reaction mixture, which reduces the enzyme activity by 50% (IC_50_). Data are presented as means ± standard deviations.

### 4.7. α-Glucosidase Inhibitory Assay

The inhibition of the α-glucosidase enzyme was determined according to the procedure described by Romeiras et al. (2023) [78]. Reaction mixtures containing 5 μL of α-glucosidase from *S. cerevisiae* (6.25 U/mL in phosphate buffer (pH 6.9, 0.1 M)), 125 μL of phosphate buffer (pH 6.9, 0.1 M), and 20 μL of the peptide fraction 2–10 kDa (concentrations up to 3.6 mg/mL) were prepared in a 96 wells microplate (Greiner Bio-One, Rainbach im Mühlkreis, Austria) and incubated for 15 min at 37 °C. Then, 20 μL of substrate solution (p-Nitrophenyl-α-D-glucopyranoside, 2.75 mM in phosphate buffer (pH 6.9, 0.1 M)) was added and the plates were incubated for an additional 15 min, at 37 °C. The reaction was stopped with 80 μL of 0.2 M Na_2_CO_3_. The absorbance of the wells was measured in a microplate reader (FLUOstar^®^ Omega Plate Reader, BMG Labtech, Ortenberg, Germany) at 405 nm. Samples’ blanks were prepared (SB) without α-glucosidase and the reaction mixture without peptide fraction was used as negative control (NC). The enzyme inhibitory rate was calculated as follows:

Inhibition (%) = [(Abs_NC_ − (Abs_sample_ − Abs_SB_) × 100/(Abs_NC_)]

Acarbose was used as positive control. Values were assessed in quadruplicate, and the results were expressed as the final concentration (μg/mL) in the reaction mixture, which reduces the enzyme activity by 20% (IC_20_). Data are presented as means ± standard deviations.

### 4.8. Statistical Analysis

The experimental data were analysed in GraphPad Prism v8.2.1. Data were expressed as mean ± standard deviation (SD), except when specified otherwise, of at least three independent experiments. When the *p*-value between two results was lower than 0.05, it was considered statistically significant.

## 5. Conclusions

In conclusion, this study demonstrates that the 2–10 kDa fraction has an antimicrobial, antioxidant, anti-inflammatory, and antidiabetic activity. Although the present study constitutes the first attempt at investigating the 2–10 kDa fraction activities, further studies are needed to confirm its efficacy and to evaluate its safety in food and health care. Overall, this research represents a significant advance in the evaluation of the functional biological properties of *S. cerevisiae* bioactive peptides.

## Figures and Tables

**Table 1:** Minimum inhibitory concentration (MIC) of 2–10 kDa fraction against the tested microorganisms.

MIC (mg/mL)				
Tested Microorganisms	2–10 kDa Fraction	Chloramphenicol ^a^	Ketoconazole ^a^	Ethanol ^b^
*C. albicans*	1.0	-	0.012	n.d
*C. krusei*	>1.0	-	0.012	n.d
*E. coli*	0.25	0.012	-	n.d
*L. monocytogenes*	0.25	0.050	-	n.d
*Salmonella* sp.	0.25	0.025	-	n.d

^a^—Positive control; ^b^—Negative control; - not tested; n.d.—not detected (MIC > 4% (*v*/*v*) of ethanol).

**Table 2:** Relative IC_50_ values (mg/mL) of peptide fraction 2–10 kDa in colorectal carcinoma (HCT116) and normal colon (coN) cell lines.

Cell Line	HCT116	coN
IC_50_ (mg/mL)	0.44 ± 0.10	0.42 ± 0.08

**Table 3:** α-Amylase and α-glucosidase inhibitory activity of peptide fraction 2–10 kDa.

	Peptide Fraction	Acarbose
α-Amylase (IC_50_ (µg/mL))	199.3 ± 0.9	11.6 ± 1.0
α-Glucosidase (IC_20_ (µg/mL))	270.6 ± 6.0	134.1 ± 4.0

## Data Availability

The data presented in this study are available in the manuscript and supplementary material.

## Supplementary Materials

The following supporting information can be downloaded at: https://www.mdpi.com/article/10.3390/antibiotics12081332/s1, Figure S1: Calibration curve and equation of DPPH test (A) and FRAP method (B). Relative antioxidant activity was expressed as percentage of absorbance decrease and subsequently calculated to equivalent content of Trolox.
