# Supplementary material for: Exploring the Multifaceted Potential of a Peptide Fraction Derived from Saccharomyces cerevisiae Metabolism: Antimicrobial, Antioxidant, Antidiabetic, and Anti-Inflammatory Properties
Source: Antibiotics (Basel). 2023 Aug 18;12(8):1332. doi: 10.3390/antibiotics12081332 (PMC10451726; doi:10.3390/antibiotics12081332)
Supplement: Supplementary file 1 [file antibiotics-12-01332-s001.zip › antibiotics-2504972-supplementary.pdf]

Supplementary material

Exploring the Multifaceted Potential of a Peptide Fraction Derived from *Saccharomyces cerevisiae* Metabolism: Antimicrobial, Antioxidant, Antidiabetic, and Anti-Inflammatory Properties

Patrícia Branco [1,2,3,*], Elisabete Muchagato Maurício [1,4,5,*], Ana Costa [2], Diogo Ventura [2], Catarina Roma-Rodrigues [6,7], Maria Paula Duarte [8], Alexandra R. Fernandes [6,7] and Catarina Prista [2]

[1] School of Engineering, Lusófona University, Campo Grande 376, 1749-024 Lisboa, Portugal
[2] Linking Landscape, Environment, Agriculture and Food (LEAF), Associated Laboratory TERRA,
Instituto Superior de Agronomia, University of Lisbon, Tapada da Ajuda, 1349-017 Lisboa, Portugal;
acs.costa@fct.unl.pt (A.C.); isa126000@isa.ulisboa.pt (D.V.); cprista@isa.utl.pt (C.P.)
[3] Unit of Bioenergy and Biorefinary, Laboratório Nacional de Energia e Geologia (LNEG,),
Estrada do Paço do Lumiar, 22, 1649-038 Lisboa, Portugal
[4] CBIOS—Universidade Lusófona's Research Center for Biosciences & Health Technologies,
Campo Grande 376, 1749-024 Lisboa, Portugal
[5] Elisa Câmara, Lda, Dermocosmética, Centro Empresarial de Talaíde, n°7 e 8,
2785-723 São Domingos de Rana, Portugal
[6] UCIBIO—Applied Molecular Biosciences Unit, Department Ciências da Vida, NOVA School of Science
and Technology, 2829-516 Caparica, Portugal; catromar@fct.unl.pt (C.R.-R.);
ma.fernandes@fct.unl.pt (A.R.F.)
[7] i4HB, Associate Laboratory—Institute for Health and Bioeconomy, Faculdade de Ciências e Tecnologia,
Universidade NOVA de Lisboa, 2829-516 Caparica, Portugal
[8] MEtRICs, Departamento de Química, NOVA School of Science and Technology|FCTNOVA,
Campus de Caparica, Universidade Nova de Lisboa, 2829-516 Caparica, Portugal;
mpcd@fct.unl.pt
* Correspondence: patricia.branco@ulusofona.pt (P.B.); elisabete.mauricio@ulusofona.pt (E.M.M.)

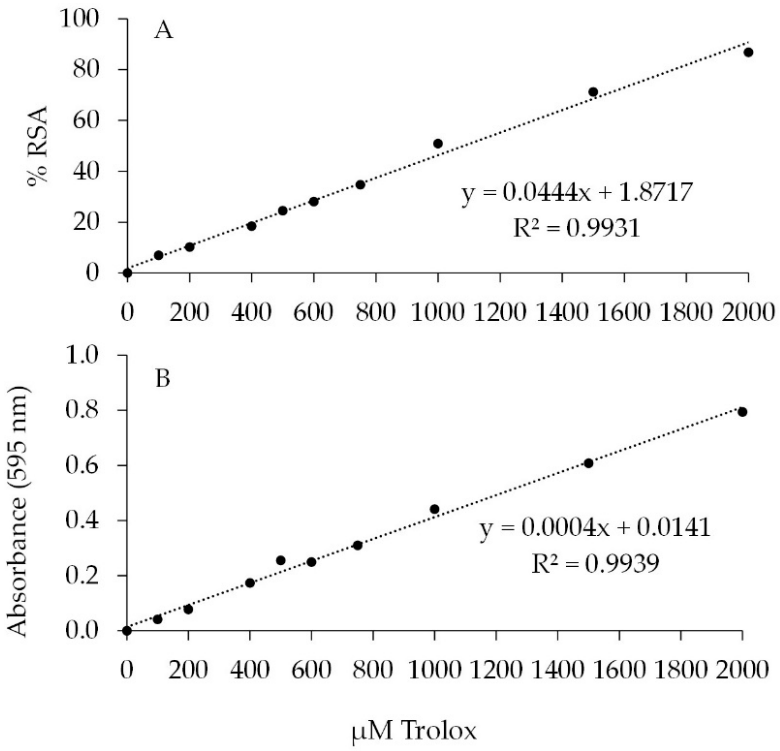

Figure S1. Calibration curve and equation of DPPH test (A) and FRAP method (B). Relative antioxidant activity was expressed as percentage of absorbance decrease and subsequently calculated to equivalent content of Trolox.
